# Monitoring chicken flock behaviour provides early warning of infection by human pathogen *Campylobacter*

**DOI:** 10.1098/rspb.2015.2323

**Published:** 2016-01-13

**Authors:** Frances M. Colles, Russell J. Cain, Thomas Nickson, Adrian L. Smith, Stephen J. Roberts, Martin C. J. Maiden, Daniel Lunn, Marian Stamp Dawkins

**Affiliations:** 1 Department of Zoology, University of Oxford, 1 South Parks Road, Oxford OX1 3TG, UK; 2 Department of Engineering Science, University of Oxford, 1 South Parks Road, Oxford OX1 3TG, UK; 3 Department of Statistics, University of Oxford, 1 South Parks Road, Oxford OX1 3TG, UK

**Keywords:** *Campylobacter*, broiler chickens, animal welfare, zoonoses, food safety

## Abstract

*Campylobacter* is the commonest bacterial cause of gastrointestinal infection in humans, and chicken meat is the major source of infection throughout the world. Strict and expensive on-farm biosecurity measures have been largely unsuccessful in controlling infection and are hampered by the time needed to analyse faecal samples, with the result that *Campylobacter* status is often known only after a flock has been processed. Our data demonstrate an alternative approach that monitors the behaviour of live chickens with cameras and analyses the ‘optical flow’ patterns made by flock movements. *Campylobacter*-free chicken flocks have higher mean and lower kurtosis of optical flow than those testing positive for *Campylobacter* by microbiological methods. We show that by monitoring behaviour in this way, flocks likely to become positive can be identified within the first 7–10 days of life, much earlier than conventional on-farm microbiological methods. This early warning has the potential to lead to a more targeted approach to *Campylobacter* control and also provides new insights into possible sources of infection that could transform the control of this globally important food-borne pathogen.

## 1. Introduction

Humans currently consume nearly 60 billion chickens a year, numerically more than any other food animal [1], and chicken production is already so efficient that modern broilers convert 3 kg of food into 2 kg of meat in a lifespan of 35 days or fewer [2]. Concurrently, there is a worldwide epidemic of human gastroenteric disease caused by *Campylobacter* (predominantly *C. jejuni* and *C. coli*) [3,4]. While these bacteria are genetically diverse, associations of particular genotypes with different host sources are stable over many decades, and attribution modelling estimates that between 58 and 78% of human disease originates from contaminated chicken meat [5].

Despite intensive efforts to improve on-farm biosecurity practice over the past decade, 71.2% of EU broiler chicken flocks remained *Campylobacter*-positive at slaughter during 2008 [6] and the incidence of human disease continues unabated [7]. This suggests that environmental contamination of growing broilers may not be the only cause of high rates of *Campylobacter* infection in chickens and that key points for intervention, for example, in breeder flocks, hatcheries or in the first week of chick life, remain unrecognized [8].

One problem in identifying these intervention points is that conventional culture-based microbiology methods are cumbersome and time-consuming, making it difficult to know at what stage birds become infected or are vulnerable to infection. On-farm methods for quickly and easily identifying when flocks become infected with *Campylobacter* could therefore be an important step in understanding the source of that infection, and so a way of helping to design interventions for living birds [9].

Although *Campylobacter* is often classified as a harmless intestinal commensal of chickens that causes a zoonotic disease in humans [7], its prevalence in chicken flocks has recently been linked to welfare [10,11], with the implication that it might also affect the behaviour and health of the birds themselves. We tested the hypothesis that flocks infected with *Campylobacter* might be distinguishable by their behaviour, thus providing an immediately available assay for infected birds while they are still alive. We used a novel and non-invasive way of monitoring the behaviour of chickens throughout their lives that involved analysing the ‘optical flow’ patterns from cameras inside broiler sheds.

‘Optical flow’ works by detecting the rate of change of brightness in different parts of a series of visual images both temporally and spatially [12,13]. It is computationally simple and does not require tagging or marking individual animals, making it ideal for long-term continuous monitoring of large groups of similar animals such as egg-laying hens [14] and broiler (meat) chickens [15–18], where optical flow is predictive of key welfare measures in such as percentage mortality and hockburn [15,16].

To test the hypothesis that optical flow analysis might also be able to detect when flocks become infected with *Campylobacter*, we collected optical flow data for 31 commercial broiler flocks. We also collected faecal samples from those same flocks and tested them for the presence of *Campylobacter* at different ages (day 21, day 28 and day 35 of age) using standard laboratory methods. We thus had a direct comparison between optical flow and testing from faecal samples.

## 2. Material and methods

### (a) Farms and birds

Data (optical flow and pathogen sampling) were collected between October 2010 and November 2014 from 31 commercial chicken flocks from three separate sites in the UK belonging to two different major producers. All flocks were of mixed sexes and were either Ross 308, Cobb 500 or a mixture of one of the two breeds. All were grown to a target final stocking density of 38 kg m^−2^. Lighting, feeding, temperature and other husbandry regimes were in accordance with the current practice recommended by the breeder companies. Optical flow data were collected only until 30 days of age, before any ‘thinning’ (early removed of a proportion of the flock). Details of farms and flocks including thinning and clearance times are shown in the electronic supplementary material, table S1.

### (b) *Campylobacter* sampling

Faecal sampling was employed to detect which flocks were shedding *Campylobacter*. Samples were collected using a combination of swabs placed over boots [19] as a person walked through the entire house when the chicks were 21 days, 28 days and 35 days of age, and fresh faecal samples that were collected concurrently on day 28 of chick age. Both boot swabs and fresh faecal samples were used to maximize the chance of recovery of the organism. The faecal samples were stored for subsequent more detailed analysis of *Campylobacter* genotypes and gut microbiota. Fabric boot swabs were placed over plastic overshoes after boot dipping to prevent any transfer of disinfectant and were pre-moistened with 20 ml buffered peptone water before use to promote faecal acquisition [19]. The wearer then followed a pre-determined zig-zag path through the entire house of roughly 100 m and (on day 28) collected faecal samples at 10 pre-determined points throughout the house. All samples were processed within 2 days of collection to ensure bacterial viability and were cultured using standard methods for both direct culture using mCCDA (PO0119 Oxoid Ltd, Basingstoke, UK) and enrichment culture using Exeter broth (Bolton Broth CM0983, defibrinated horse blood SR0050 and *Campylobacter* growth supplement SR0232, Oxoid Ltd, Basingstoke, UK; and Exeter *Campylobacter* enrichment-selective supplement SV59, Mast Group, Bootle, UK) and sub-culture onto mCCDA. Faecal material was loosened from the boot swabs by adding 50 ml of phosphate-buffered saline and placing in a stomacher for 30 seconds, prior to innoculating 20 µl of the resulting suspension onto mCCDA and 1 : 10 v v^−1^ into Exeter broth. All culture media were incubated at 42°C for 48 h, using a microaerobic atmosphere for solid agar plates, and a small air space for the broths. Presumptive *Campylobacter* isolates identified by characteristic colony appearance were sub-cultured onto blood agar (PB0122, Oxoid Ltd) and incubated at 42°C for a further 48 h. The identity of *Campylobacter* isolates was then confirmed by characteristic Gram-negative small curved rod appearance, and positive oxidase and catalase reactions.

### (c) Definition of *Campylobacter*-positive and -negative flocks

We originally collected data from 51 flocks from four sites, but then applied strict criteria for a flock to be included in the analysis, leaving only 31/51 (61%) flocks that met these criteria (23 from company 1 and 8 from company 2, and from only two-thirds farms of company 2). We only included flocks that had been tested on at least two separate days. Negative flocks were defined as those that were microbiologically negative at 35 days and had not tested positive on any previous sampling days. Positive flocks were defined as those that tested positive at any time and were not tested as negative subsequent to that test. Results of sampling, flock status and dates placed are given in electronic supplementary material, table S2.

### (d) Cameras and recording equipment

For farm 1, the behaviour of the broiler flocks was recorded using waterproof and custom-built Logitech C120 web cameras, connected (two cameras/unit) to a small form-factor industrial PC (Fit-PC2, Anders Electronics plc, London, UK) enclosed in a protective waterproof covering as described in more detail elsewhere [13,14]. Two units (four cameras per house) were installed. For farms 2–4, the equipment was updated and the software rewritten for ruggedized smartphones (CAT B15, Caterpillar Inc., Illinois, USA) running Android v. 4.0.4. In both cases, the cameras (or smartphones) were mounted at a height of 2.0 ± 0.1 m. Both had a focal length (35 mm equivalent) of approximately 25 mm, giving a ‘wide angle’ field of view of approx. 3 × 3 m of floor area. They were connected to a mains power supply and positioned so that the field of view contained less than 10% of static objects such as feeders, drinkers and house uprights. Both systems recorded with a frame rate of four frames per second. The two systems were calibrated with test runs of the same data and shown to give similar results (*r*^2^ for kurtosis = 0.84, *n* = 20, *p* < 0.0001, Pearson correlation), but to ensure that the change of equipment did not affect the results, data from a given flock were compared only with data collected on the same system. Cameras were installed before the chicks arrived and left running until day 35. Day 1 data were not used as the chicks were clearly unsettled on arrival, and data for company 2 days 1–5 were not available due to camera faults.

### (e) Optical flow

Optical flow analysis involves detecting the rate of change of brightness in each area of an image frame both temporally and spatially. These changes are combined to give an estimate of local velocity vectors [10,11]. Data were collected at four frames a second between 08.00 and 20.00 each day. Each image frame on a video file was divided into 320 × 240 pixel images divided into 1200 (i.e. 40 × 30) 8-by-8 pixel blocks and optical flow statistics (mean, variance, skew, kurtosis) calculated every 15 min (all 4 Hz frames) [15,16]. For each flock, daily means for each statistic were calculated by taking the average of all four cameras, originally using MATLAB and subsequently a combination of Python and C code for improved processing efficiency.

### (f) Statistical analysis

The data were analysed with multi-level models. The explanatory variables were the presence/absence of *Campylobacter* and chick age in days expressed in terms of orthogonal polynomials up to and including fourth order. Interaction terms were included in order to account for profile differences between *Campylobacter*-positive and -negative flocks. The same methodology was used to perform separate analyses on the two optical flow descriptors, mean and kurtosis, that had previously been shown to be of particular importance to health and welfare outcomes [15,16]. For both mean and kurtosis, the measure used was average daily value (over the 12 h period 08.00–20.00). Diagnostics were carried out on the fitted model to confirm normality and homoscedasticity.

#### (i) Company

Because housing, management and recording equipment differed between companies, data from each company were analysed separately. This means that the comparison between *Campylobacter*-positive and -negative flocks was based on flocks belonging to the same company. There was not enough data to separate farm and company effects.

#### (ii) Temperature

To take account of possible effects of variation in external temperature at different times of year, we obtained minimum and maximum monthly temperature readings from the UK Met Office (http://www.metoffice.gov.uk/climate/uk/) using data from the weather station closest to each farm (electronic supplementary material, table S2). The mid-range of temperatures for the month in which the chicks were hatched was entered into the multi-level model.

#### (iii) *Campylobacter* and age

For each company, for both daily mean and daily kurtosis, exploratory plots showed that the 30-day profiles had three turning points, and so orthogonal polynomials up to and including fourth order were fitted.

Modelling used the nlme library from the R statistical package [20] and *R*^2^-values based on the likelihood ratio were used to give an indication of goodness of fit [21].

Taking ‘flock’ as a random variable (i.e. assuming that the flocks observed were a random sample of all flocks from that company and would naturally vary in movement), it was possible to incorporate random effects into the coefficients of the order 1 (change of mean or kurtosis) and order 2 (rate of change) coefficients. Including these random effects in the model resulted in a highly significant increase in the log likelihood (*p* < 0.001). Using this best-fit model, robust 95% bootstrap confidence envelopes were obtained for all of the plotted curves. For each curve, over 1000 bootstrap replicates were obtained at each time point [22].

## 3. Results

**Figure 1.**
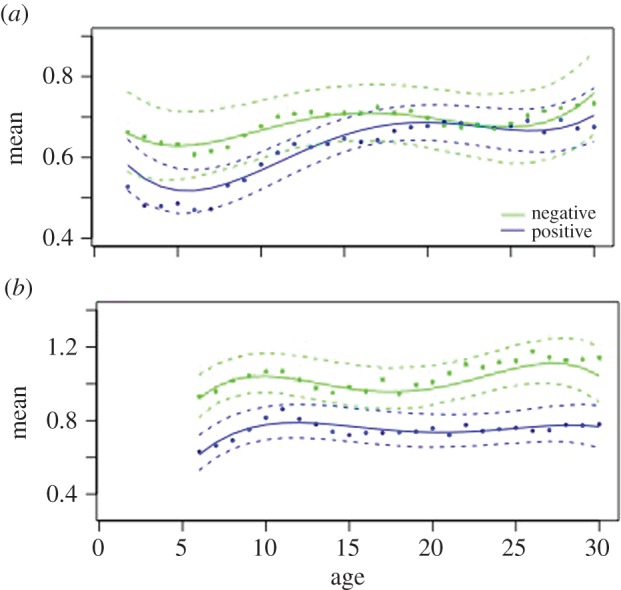
Daily mean optical flow for (*a*) company 1 and (*b*) company 2 for *Campylobacter*-positive (blue) and *Campylobacter*-negative flocks (green). The solid lines show the best-fit fourth-order model for daily mean values (daily means of mean optical flow). The dots represent the actual observed daily mean values and the dashed lines are the 95% confidence limits for bootstrapped model data. The *x*-axis is the age in days.

*Campylobacter*-positive flocks showed lower mean optical flow than flocks not detected as shedding *Campylobacter*, as early as the first 10 days of life (figure 1*a*,*b* and table 1). For company 1, the shapes of the two age curves (positive and negative) are similar and are distinguished by location and depth of the turning points. The model fit is very close, as indicated by the fact that almost all the points are inside the bootstrap confidence limits and the *R*^2^-value = 0.8575. For company 2, the same lower value of mean optical flow for *Campylobacter*-positive flocks is apparent (figure 1*b* and table 1), and this difference between flocks of different *Campylobacter* status is maintained throughout life. Once again the model fit is good (*R*^2^ = 0.8378).

**Table 1.** Coefficients of fitted model for daily mean optical flow.

	coefficient	value	d.f.	*t*-value	*p*-value
company 1	intercept	0.8769	532	12.1832	0.0000
	*Camp*Pos	−0.0917	18	−1.0789	0.2949
	temperature	0.0079	18	1.4207	0.1725
	oAge1*Camp*Pos	0.1717	532	1.0858	0.2781
	oAge2*Camp*Pos	−0.0188	532	−0.3002	0.7642
	oAge3*Camp*Pos	−0.1257	532	−5.6008	0.0000
	oAge4*Camp*Pos	0.0168	532	0.7506	0.4532
company 2	intercept	−1.4182	158	−0.1893	0.8501
	*Camp*Pos	0.6668	4	0.1624	0.8789
	temperature	−0.1523	4	−0.2271	0.8315
	*o*Age1*Camp*Pos	−0.1162	158	−0.5932	0.5539
	*o*Age2*Camp*Pos	−0.0770	158	−0.3655	0.7152
	oAge3*Camp*Pos	−0.0838	158	−0.8029	0.4233
	oAge4*Camp*Pos	0.2153	158	3.1551	0.0019

**Figure 2.**
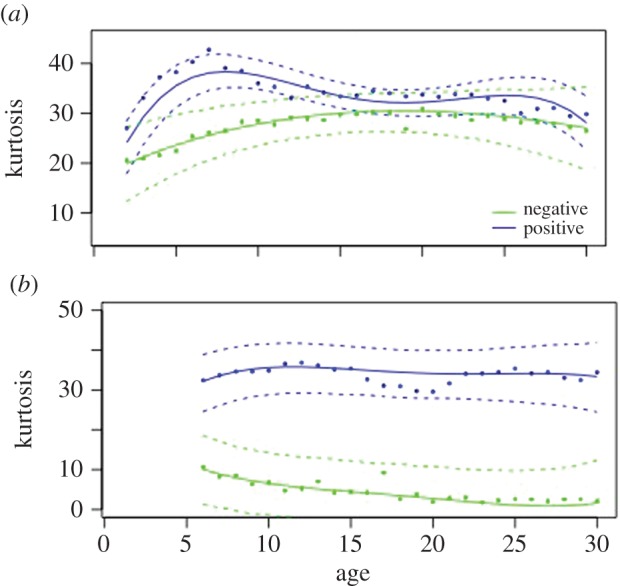
Daily kurtosis optical flow for (*a*) company 1 and (*b*) company 2 for *Campylobacter*-positive (blue) and *Campylobacter*-negative flocks (green). The solid lines show the best-fit fourth-order model for daily mean values (daily means of kurtosis optical flow). The dots represent the actual observed daily mean values and the dashed lines are the 95% confidence limits for boostrapped model data. The *x*-axis is the age in days.

For kurtosis of optical flow, the effects are even more apparent, with *Campylobacter*-positive flocks showing consistently higher values than negative flocks (figure 2). For company 1, there are significant contrasts between flocks with different disease status in both location and shape. Being *Campylobacter*-positive produces an upward shift in kurtosis of 5.44 and highly significant interactions between disease and the third and fourth polynomials (table 2). Model fit is good (*R*^2^ = 0.9247). For company 2, an even greater upward shift is apparent (26.81) and the fit is even closer (*R*^2^ = 0.9879).

**Table 2.** Coefficients of fitted model for daily kurtosis optical flow.

	coefficient	value	d.f.	*t*-value	*p*-value
company 1	intercept	20.9575	532	6.9515	0.0000
	*Camp*Pos	5.0119	18	2.1215	0.0473
	temperature	0.0616	18	1.6869	0.1080
	oAge1*Camp*Pos	−11.6433	532	−0.8142	0.4159
	oAge2*Camp*Pos	−1.0755	532	−0.1761	0.8612
	oAge3*Camp*Pos	11.7441	532	6.6239	0.0000
	oAge4*Camp*Pos	−12.8840	532	−7.3100	0.0000
company 2	intercept	5.8956	158	1.3675	0.1734
	*Camp*Pos	26.8173	6	4.9176	0.0027
	temperature	0.0104	18	1.7460	0.8101
	oAge1*Camp*Pos	38.8721	158	2.0434	0.0427
	oAge2*Camp*Pos	−27.3677	158	−2.6389	0.0091
	oAge3*Camp*Pos	15.7648	158	3.6063	0.0004
	oAge4*Camp*Pos	−8.0803	158	−2.8023	0.0054

Importantly, temperature had no significant effects on the differences between *Campylobacter*-positive and -negative flocks in either mean or kurtosis of optical flow, and there were no significant interactions between the presence/absence of *Campylobacter* and temperature (table 2).

## 4. Discussion

Our results provide statistical evidence of a link between broiler chicken flock behaviour and *Campylobacter* status, as early as the first 10 days of life. Flocks shedding *Campylobacter* have a lower mean optical flow (less average movement) and higher kurtosis (less uniform movement) than flocks not detected as shedding *Campylobacter*. This link is independent of external temperature and cannot be explained by a direct effect of temperature on the behaviour of a flock. Thus, although the incidence of *Campylobacter* may be higher with warmer temperatures, the *differences* in optical flow between positive and negative flocks, in both mean and kurtosis, are independent of temperature.

Our results are compatible with the growing evidence that *Campylobacter* may not be just a harmless commensal member of the chicken gut microbiota, but is associated with reduction in the health of the chickens [7]. Reduced growth rate in free-range chickens is associated with increased diversity of *Campylobacter* genotypes [23], and where chickens show a strong inflammatory response to *Campylobacter,* this can lead to diarrhoea, poor-quality wet litter, and damage to birds' feet and legs [7]. Greater susceptibility to *Campylobacter* has also been associated with changes in the gut microbiota of chickens, humans and mice, while vibrionic hepatitis (spotty liver disease) in chickens is also strongly linked with *Campylobacter* [24–26].

What is not clear, however, is whether the lower mean and higher kurtosis of optical flow associated here with *Campylobacter* infection is the result of a direct effect of *Campylobacter* on chicken behaviour or whether the optical flow is detecting other signs of reduced welfare (such as poorer walking ability) that indicate a general reduction in the birds' overall health. We also do not know whether the optical flow differences that were apparent in very young birds (less than 10 days old) were due to their already being infected but not shedding sufficiently for infection to be detected or whether they were still uninfected but had slightly reduced overall condition that would make them vulnerable to later infection. However, whether *Campylobacter* is affecting chickens directly or is an indicator of other predisposing factors, detecting its presence could be a valuable aid to healthy flock management for producers. Detecting health and welfare issues at an early stage provides opportunities for interventions before commercially damaging consequences occur.

Our results also suggest new directions in identifying causes of *Campylobacter* infection of chickens. First, although direct vertical transmission of *Campylobacter* between parent and progeny flocks is not widely regarded as important on the grounds that the bacteria are only rarely isolated from eggs, our finding that susceptible flocks are identifiable when the flocks are only 7–10 days old points to the possible importance of transgenerational influences, or at least to particular susceptibility in early life [27]. Second, closer attention should be paid to the hypothesis that there may be inherent differences between broiler flocks (e.g. number of parent flocks supplying eggs, breed) or the management of flocks in the first week of life that contribute to the development of *Campylobacter* infection. Stress increases the uptake of *Campylobacter* in the gut epithelium [2,28], and chicks that experience temperature and humidity levels outside recommended boundaries in their first 7 days have increased risk of mortality and leg health problems in later life [29,30]. Reduction of stress through careful management of the environment experienced by young chicks may be an important potential way of combating *Campylobacter* infection.

## 5. Conclusion

Systematic use of optical flow information has the potential to make a major impact on the management of commercial chicken flocks, to the benefit of producers, consumers and the birds themselves. Farm managers able to access such information in real time would have an early warning of which of their flocks were most at risk of health and welfare problems, enabling them to intervene before these became serious and helping them to produce higher-quality, healthier food with better welfare.

## Supplementary Material

FARMTABLE1.pdf

## Supplementary Material

FARMTABLE2.pdf

## Data accessibility

The data supporting this article are available as electronic supplementary material.
