# Supplementary material for: Monitoring chicken flock behaviour provides early warning of infection by human pathogen Campylobacter
Source: Proc Biol Sci. 2016 Jan 13;283(1822):20152323. doi: 10.1098/rspb.2015.2323 (PMC4721092; doi:10.1098/rspb.2015.2323)
Supplement: FARMTABLE1.pdf [file rspb20152323supp1.pdf]

Table 1. Farm information
Data was collected from 4 farms but none of the flocks from Farm 4 met the criteria for inclusion in the analysis

Farm	Co. no.	No.houses	House size	No.drinkers	No.feeders	No. chicks	Thin age	Final age
1	1	6	1670m^2	1735	485	33,000	32/33d	35-46d
2	2	3	1046m2	1650	300	22,000	32d	38d
3	2	4	642.6m2	1035	156	13,750	33d	37d
4	2	4	1046m2	1650	288	22,000	33d	37d
