# Supplementary material for: Monitoring chicken flock behaviour provides early warning of infection by human pathogen Campylobacter
Source: Proc Biol Sci. 2016 Jan 13;283(1822):20152323. doi: 10.1098/rspb.2015.2323 (PMC4721092; doi:10.1098/rspb.2015.2323)
Supplement: FARMTABLE2.pdf [file rspb20152323supp2.pdf]

DAWKINS et al. OPTICAL FLOW TEMPERATURE AND FAECAL SAMPLING DATA

Table 2. Decscription of flocks observed (n=31)

Criteria for inclusion in the analysis are described in the Methods section

The optical flow values refer to daily means for each descriptor.

Flock numbers refer to our private flock codes and have no further significance

Date refers to chick placement date; age is give in days

								Faecal				Temp	
Flock	Date	Co.	Farm	House	Age	Mean	Kurtosis	day21	day28	day35	Result	tmax	tmin
9	4.1.11	1	1	2	1	2.48	36.84	Neg	No	Pos	POS	6.9	1.3
					2	0.56	39.99						
					3	0.50	41.82						
					4	0.48	43.33						
					5	0.57	37.59						
					6	0.62	35.79						
					7	0.63	33.23						
					8	0.68	32.17						
					9	0.66	34.32						
					10	0.66	37.68						
					11	0.69	35.95						
					12	0.68	33.21						
					13	0.70	33.61						
					14	0.73	31.73						
					15	0.77	30.65						
					16	0.73	30.41						
					17	0.74	31.05						
					18	0.76	30.17						
					19	0.70	27.87						
					20	0.68	28.73						
					21	0.69	28.99						
					22	0.67	29.47						
					23	0.69	29.23						
					24	0.68	28.78						
					25	0.71	26.55						
					26	0.75	26.73						
					27	0.75	26.97						
					28	0.77	26.79						
					29	0.77	27.24						
					30	0.84	26.47						

								Faecal samples				Temp	
Flock	Date	Co.	Farm	House	Age	Mean	Kurtosis	day21	day28	day35	Result	tmax	tmin
10	4.1.11	1	1	3	1	0.89	3.55	Neg	No	Pos	POS	6.9	1.3
					2	0.64	15.16						
					3	0.47	29.61						
					4	0.46	40.39						
					5	0.41	43.09						

					6	0.38	44.63						
					7	0.35	48.86						
					8	0.34	43.67						
					9	0.38	39.94						
					10	0.47	37.08						
					11	0.51	33.40						
					12	0.55	31.84						
					13	0.56	31.75						
					14	0.53	31.18						
					15	0.61	29.21						
					16	0.57	29.55						
					17	0.64	28.65						
					18	0.65	29.56						
					19	0.57	31.00						
					20	0.61	29.35						
					21	0.71	28.68						
					22	0.65	28.83						
					23	0.64	29.19						
					24	0.64	29.06						
					25	0.66	28.50						
					26	0.66	28.25						
					27	0.67	27.84						
					28	0.72	27.83						
					29	0.73	27.12						
					30	0.70	26.70						

								Faecal samples				Temp	
Flock	Date	Co.	Farm	House	Age	Mean	Kurtosis	day21	day28	day35	Result	tmax	tmin
11	4.1.11	1	1	5	1	0.48	32.38	Neg	No	Neg	NEG	6.9	1.3
					2	0.34	50.65						
					3	0.34	51.52						
					4	0.34	49.75						
					5	0.39	46.62						
					6	0.42	42.18						
					7	0.49	40.43						
					8	0.49	38.80						
					9	0.54	36.66						
					10	0.57	33.33						
					11	0.60	32.94						
					12	0.67	33.16						
					13	0.67	31.93						
					14	0.60	31.16						
					15	0.56	32.18						
					16	0.59	31.58						
					17	0.66	31.88						
					18	0.68	34.03						

					19	0.75	34.26						
					20	0.75	33.98						
					21	0.66	31.62						
					22	0.60	29.86						
					23	0.61	28.33						
					24	0.62	28.69						
					25	0.67	27.67						
					26	0.69	25.64						
					27	0.71	23.58						
					28	0.69	24.53						
					29	0.71	24.02						
					30	0.74	22.60						

								Faecal samples				Temperature	
Flock	Date	Co.	Farm	House	Age	Mean	Kurtosis	day21	day28	day35	Result	tmax	tmin
12	4.1.11	1	1	6	1	0.91	20.77	Neg	No	Neg	NEG	6.9	1.3
					2	0.61	40.39						
					3	0.64	38.86						
					4	0.61	37.41						
					5	0.63	35.75						
					6	0.59	36.85						
					7	0.63	34.39						
					8	0.64	31.50						
					9	0.65	30.52						
					10	0.68	29.72						
					11	0.69	28.10						
					12	0.71	29.15						
					13	0.72	28.25						
					14	0.80	24.80						
					15	0.79	25.03						
					16	0.78	25.24						
					17	0.81	24.78						
					18	0.76	26.20						
					19	0.77	25.25						
					20	0.77	25.77						
					21	0.77	25.23						
					22	0.81	24.30						
					23	0.79	23.78						
					24	0.79	23.38						
					25	0.82	23.66						
					26	0.94	18.44						
					27	0.88	19.91						
					28	0.87	21.47						
					29	0.86	21.20						
					30	0.85	21.91						

								Faecal samples				Temp	
Flock	Date	Co.	Farm	House	Age	Mean	Kurtosis	day21	day28	day35	Result	tmax	tmin
17	####	1	1	2	1	1.28	1.57	Neg	Pos	Pos	POS	18.6	6.6
					2	0.51	23.67						
					3	0.42	21.44						
					4	0.45	39.43						
					5	0.43	42.32						
					6	0.38	42.86						
					7	0.31	60.79						
					8	0.38	58.64						
					9	0.33	60.91						
					10	0.45	47.78						
					11	0.45	49.48						
					12	0.59	35.66						
					13	0.48	45.26						
					14	0.49	40.54						
					15	0.53	41.90						
					16	0.50	44.31						
					17	0.48	44.57						
					18	0.48	47.48						
					19	0.55	38.50						
					20	0.55	39.22						
					21	0.59	40.15						
					22	0.55	38.75						
					23	0.65	37.20						
					24	0.54	37.31						
					25	0.56	38.20						
					26	0.59	34.06						
					27	0.53	35.31						
					28	0.54	33.79						
					29	0.59	34.29						
					30	0.57	33.96						

								Faecal samples				Temp	
Flock	Date	Co.	Farm	House	Age	Mean	Kurtosis	day21	day28	day35	Result	tmax	tmin
18	####	1	1	3	1	1.32	1.56	Neg	Neg	Pos	POS	18.6	6.6
					2	0.55	27.59						
					3	0.39	27.73						
					4	0.38	41.07						
					5	0.41	43.26						
					6	0.38	44.11						
					7	0.45	44.82						
					8	0.49	43.54						
					9	0.48	37.62						
					10	0.52	36.84						
					11	0.51	36.27						

					12	0.56	34.92						
					13	0.57	34.43						
					14	0.59	34.14						
					15	0.56	32.25						
					16	0.60	32.66						
					17	0.61	33.12						
					18	0.67	34.01						
					19	0.67	34.43						
					20	0.62	34.92						
					21	0.63	34.11						
					22	0.72	36.44						
					23	0.67	34.43						
					24	0.61	34.34						
					25	0.68	34.22						
					26	0.70	31.74						
					27	0.61	33.03						
					28	0.58	31.92						
					29	0.56	30.92						
					30	0.59	30.40						

								Faecal samples				Temp	
Flock	Date	Co.	Farm	House	Age	Mean	Kurtosis	day21	day28	day35	Result	tmax	tmin
19	####	1	1	5	1	0.48	39.48	Neg	Neg	Pos	POS	18.6	6.6
					2	0.44	47.94						
					3	0.37	49.33						
					4	0.34	47.70						
					5	0.33	48.91						
					6	0.35	46.92						
					7	0.42	49.74						
					8	0.44	48.05						
					9	0.50	45.80						
					10	0.48	41.04						
					11	0.54	38.83						
					12	0.59	33.95						
					13	0.59	34.41						
					14	0.59	32.83						
					15	0.62	32.64						
					16	0.65	33.96						
					17	0.65	34.81						
					18	0.68	34.51						
					19	0.74	35.15						
					20	0.80	36.06						
					21	0.75	34.33						
					22	0.73	35.74						
					23	0.70	33.90						
					24	0.83	37.39						

					25	0.71	33.66						
					26	0.71	34.85						
					27	0.64	29.64						
					28	0.68	30.80						
					29	0.66	28.77						
					30	0.64	29.10						

								Faecal samples				Temp	
Flock	Date	Co.	Farm	House	Age	Mean	Kurtosis	day21	day28	day35	Result	tmax	tmin
20	####	1	1	6	1	0.52	34.06	Neg	Neg	Pos	POS	18.6	6.6
					2	0.51	38.16						
					3	0.50	36.88						
					4	0.47	37.49						
					5	0.42	41.36						
					6	0.47	37.99						
					7	0.44	38.32						
					8	0.49	36.50						
					9	0.47	38.17						
					10	0.49	33.83						
					11	0.53	34.92						
					12	0.53	34.92						
					13	0.53	34.92						
					14	0.53	34.92						
					15	0.53	34.92						
					16	0.53	34.92						
					17	0.53	34.92						
					18								
					19								
					20								
					21								
					22								
					23								
					24								
					25								
					26								
					27								
					28								
					29								
					30								

								Faecal samples				Temp	
Flock	Date	Co.	Farm	House	Age	Mean	Kurtosis	day21	day28	day35	Result	tmax	tmin
21	####	1	1	2	1	1.49	3.51	Pos	Pos	Pos	POS	17.5	8.9
					2	0.47	25.49						
					3	0.30	53.84						
					4	0.35	48.41						

					5	0.39	44.73						
					6	0.33	51.39						
					7	0.26	55.06						
					8	0.32	47.00						
					9	0.36	43.45						
					10	0.45	35.86						
					11	0.50	36.22						
					12	0.49	35.65						
					13	0.52	34.65						
					14	0.53	35.85						
					15	0.53	33.89						
					16	0.48	34.72						
					17	0.51	33.95						
					18	0.55	32.23						
					19	0.61	30.21						
					20	0.64	29.67						
					21	0.59	30.60						
					22	0.65	29.65						
					23	0.66	28.55						
					24	0.64	27.34						
					25	0.62	27.53						
					26	0.61	26.55						
					27	0.62	28.62						
					28	0.68	27.60						
					29	0.67	27.97						
					30								

								Faecal samples				Temp	
Flock	Date	Co.	Farm	House	Age	Mean	Kurtosis	day21	day28	day35	Result	tmax	tmin
22	####	1	1	3	1	1.30	1.52	Pos	Pos	Pos	POS	17.5	8.9
					2	0.67	13.06						
					3	0.39	31.71						
					4	0.40	40.47						
					5	0.41	44.72						
					6	0.38	48.41						
					7	0.39	47.06						
					8	0.40	45.60						
					9	0.40	47.51						
					10	0.50	44.01						
					11	0.57	41.42						
					12	0.55	40.10						
					13	0.64	39.34						
					14	0.59	35.67						
					15	0.57	34.80						
					16	0.60	29.01						
					17	0.63	30.69						

					18	0.64	31.01						
					19	0.67	29.76						
					20	0.70	31.23						
					21	0.71	29.38						
					22	0.68	30.82						
					23	0.72	31.75						
					24	0.67	28.66						
					25	0.63	27.31						
					26	0.71	26.31						
					27	0.68	29.11						
					28	0.76	26.30						
					29	0.83	26.36						
					30	0.66	27.22						

								Faecal samples				Temp	
Flock	Date	Co.	Farm	House	Age	Mean	Kurtosis	day21	day28	day35	Result	tmax	tmin
23	####	1	1	5	1	0.38	34.56	Pos	Pos	Pos	POS	17.5	8.9
					2	0.28	24.16						
					3	0.33	35.05						
					4	0.29	41.58						
					5	0.37	45.53						
					6	0.34	45.03						
					7	0.39	47.24						
					8	0.41	51.05						
					9	0.43	51.41						
					10	0.46	46.29						
					11	0.50	43.37						
					12	0.53	40.28						
					13	0.64	36.50						
					14	0.64	40.82						
					15	0.60	37.60						
					16	0.61	39.43						
					17	0.63	41.23						
					18	0.67	39.13						
					19	0.63	36.66						
					20	0.65	40.52						
					21	0.73	38.74						
					22	0.71	39.13						
					23	0.71	45.42						
					24	0.73	40.85						
					25	0.75	40.05						
					26	1.52	17.44						
					27	1.12	24.91						
					28	0.75	34.03						
					29	0.58	29.38						
					30	0.80	35.37						

								Faecal samples				Temp	
Flock	Date	Co.	Farm	House	Age	Mean	Kurtosis	day21	day28	day35	Result	tmax	tmin
24	####	1	1	6	1	0.43	23.08	Pos	Pos	Pos	POS	17.5	8.9
					2	0.37	42.09						
					3	0.30	53.94						
					4	0.26	63.83						
					5	0.29	57.02						
					6	0.29	62.83						
					7	0.29	61.23						
					8	0.34	54.72						
					9	0.35	50.82						
					10	0.46	45.05						
					11	0.52	41.20						
					12	0.52	43.79						
					13	0.57	43.05						
					14	0.53	44.52						
					15	0.60	40.69						
					16	0.54	40.35						
					17	0.48	41.01						
					18	0.54	37.08						
					19	0.59	37.05						
					20	0.59	37.03						
					21	0.58	35.35						
					22	0.59	35.72						
					23	0.57	33.91						
					24	0.60	31.75						
					25	0.59	31.58						
					26	0.61	30.31						
					27	0.58	32.47						
					28	0.56	32.81						
					29	0.57	32.76						
					30	0.59	31.62						

								Faecal samples				Temp	
Flock	Date	Co.	Farm	House	Age	Mean	Kurtosis	day21	day28	day35	Result	tmax	tmin
39	31.3.1	2	2	1	1			No	Neg	Neg	NEG	12.3	3.5
					2								
					3								
					4								
					5								
					6	1.12	28.26						
					7	1.13	22.47						
					8	1.18	20.72						
					9	1.15	16.79						
					10	1.19	16.98						

					11	1.19	12.60						
					12	1.16	10.82						
					13	1.13	14.14						
					14	1.13	7.39						
					15	1.17	8.23						
					16	1.14	5.15						
					17	1.15	13.09						
					18	1.10	1.23						
					19	1.12	5.45						
					20	1.10	2.75						
					21	1.22	2.91						
					22	1.22	4.46						
					23	1.24	2.23						
					24	1.23	3.22						
					25	1.25	3.42						
					26	1.29	3.41						
					27	1.25	2.98						
					28	1.28	2.91						
					29	1.26	3.44						
					30	1.25	3.07						

								Faecal samples				Temp	
Flock	Date	Co.	Farm	House	Age	Mean	Kurtosis	day21	day28	day35	Result	tmax	tmin
40	31.3.1	2	2	2	1			No	Neg	Neg	NEG	12.3	3.5
					2								
					3								
					4								
					5								
					6	0.90	2.46						
					7	0.93	1.16						
					8	0.95	4.11						
					9	1.03	1.30						
					10	1.06	2.35						
					11	1.04	0.99						
					12	0.95	4.39						
					13	0.88	6.30						
					14	0.85	4.31						
					15	0.92	3.95						
					16	0.87	6.50						
					17	0.90	5.27						
					18	0.90	5.73						
					19	0.87	2.13						
					20	0.92	1.16						
					21	0.89	2.85						
					22	0.99	1.51						
					23	0.94	1.27						

					24	1.01	1.37						
					25	1.00	1.88						
					26	1.07	1.66						
					27	1.03	1.25						
					28	0.98	2.18						
					29	1.01	1.74						
					30	1.03	1.11						

								Faecal samples				Temp	
Flock	Date	Co.	Farm	House	Age	Mean	Kurtosis	day21	day28	day35	Result	tmax	tmin
41	31.3.1	2	2	3	1	1.75	46.22	No	Neg	Neg	NEG	12.3	3.5
					2	0.77	1.66						
					3	0.71	1.91						
					4	0.70	1.61						
					5	0.72	2.80						
					6	0.77	1.09						
					7	0.81	1.18						
					8	0.92	0.68						
					9	0.95	1.02						
					10	0.95	0.98						
					11	0.98	0.49						
					12	0.95	0.55						
					13	0.92	0.56						
					14	0.88	0.98						
					15	0.86	1.15						
					16	0.86	1.15						
					17								
					18	0.83	0.96						
					19								
					20								
					21								
					22								
					23								
					24								
					25								
					26								
					27								
					28								
					29								
					30								

								Faecal samples				Temp	
Flock	Date	Co.	Farm	House	Age	Mean	Kurtosis	day21	day28	day35	Result	tmax	tmin
42	16.5.1	2	2	1	1	0.86	6.51	Neg	Neg	Pos	POS	17.1	8.8
					2	0.85	14.74						
					3	0.82	16.95						

					4	0.83	16.81						
					5	0.84	15.47						
					6	0.87	16.87						
					7	0.88	18.35						
					8	0.91	19.43						
					9	0.94	20.76						
					10	0.97	21.71						
					11	0.98	25.30						
					12	0.90	24.50						
					13	0.92	24.12						
					14	0.51	23.09						
					15	0.86	25.47						
					16	0.85	28.48						
					17	0.85	27.97						
					18	0.85	27.34						
					19	0.82	25.57						
					20	0.89	23.78						
					21	0.86	27.30						
					22	0.89	29.08						
					23	0.84	27.29						
					24	0.85	27.83						
					25	0.86	29.14						
					26	0.87	27.81						
					27	0.87	28.51						
					28	0.91	27.16						
					29	0.93	25.82						
					30	0.99	26.18						

								Faecal samples				Temp	
Flock	Date	Co.	Farm	House	Age	Mean	Kurtosis	day21	day28	day35	Result	tmax	tmin
43	16.5.1	2	2	2	1	0.65	25.41	Neg	Pos	Pod	POS	17.1	8.8
					2	0.69	36.85						
					3	0.64	38.81						
					4	0.60	34.24						
					5	0.61	33.37						
					6	0.60	31.63						
					7	0.62	33.76						
					8	0.66	33.90						
					9	0.69	34.11						
					10	0.71	36.82						
					11	0.76	35.39						
					12	0.68	37.31						
					13	0.66	35.74						
					14	0.67	34.44						
					15	0.66	34.61						
					16	0.67	35.36						

					17	0.65	34.02						
					18	0.63	32.82						
					19	0.63	32.08						
					20	0.64	32.96						
					21	0.61	34.90						
					22	0.63	35.34						
					23	0.67	34.65						
					24	0.67	34.02						
					25	0.69	35.45						
					26	0.69	35.01						
					27	0.70	35.77						
					28	0.75	36.01						
					29	0.76	35.80						
					30	0.81	35.43						

								Faecal samples				Temp	
Flock	Date	Co.	Farm	House	Age	Mean	Kurtosis	day21	day28	day35	Result	tmax	tmin
44	16.5.1	2	2	3	1	0.65	18.35	Neg	Pos	Pos	POS	17.1	8.8
					2	0.64	25.91						
					3	0.60	26.93						
					4	0.58	26.65						
					5	0.59	25.73						
					6	0.59	27.26						
					7	0.65	27.49						
					8	0.67	28.81						
					9	0.75	29.10						
					10	0.81	28.91						
					11	0.98	23.43						
					12	0.77	34.79						
					13	0.76	34.93						
					14	0.73	32.63						
					15	0.69	34.43						
					16	0.68	34.15						
					17	0.70	31.22						
					18	0.73	32.65						
					19	0.76	31.60						
					20	0.74	32.01						
					21	0.69	32.73						
					22	0.70	34.74						
					23	0.69	32.94						
					24	0.73	32.07						
					25	0.74	32.90						
					26	0.74	32.64						
					27	0.74	33.38						
					28	0.74	31.45						
					29	0.75	31.22						

					30	0.75	30.68						

								Faecal samples				Temp	
Flock	Date	Co.	Farm	House	Age	Mean	Kurtosis	day21	day28	day35	Result	tmax	tmin
50	6.6.14	2	3	1	1	0.63	30.35	Pos	No	Pos	POS	20.7	11
					2	0.56	35.69						
					3	0.56	38.36						
					4	0.51	38.17						
					5	0.54	41.88						
					6	0.55	42.28						
					7	0.59	43.17						
					8	0.62	44.56						
					9	0.71	42.74						
					10	0.81	41.85						
					11	0.84	43.62						
					12	0.87	43.86						
					13	0.79	43.78						
					14	0.70	43.31						
					15	0.71	41.39						
					16								
					17								
					18								
					19								
					20								
					21								
					22	0.82	34.97						
					23	0.71	37.61						
					24	0.71	41.26						
					25	0.75	40.53						
					26	0.67	38.16						
					27	0.71	37.40						
					28	0.71	34.36						
					29	0.70	33.10						
					30	0.68	37.55						

								Faecal samples				Temp	
Flock	Date	Co.	Farm	House	Age	Mean	Kurtosis	day21	day28	day35	Result	tmax	tmin
51	6.6.14	2	3	2	1	0.57	34.87	Pos	Pos	Pos	POS	20.7	11
					2	0.53	27.46						
					3	0.55	39.20						
					4	0.51	39.98						
					5	0.51	42.34						
					6	0.53	44.03						
					7	0.57	45.70						
					8	0.60	46.38						
					9	0.66	46.38						

					10	0.78	45.04						
					11	0.86	41.99						
					12	0.84	43.82						
					13	0.77	42.29						
					14	0.70	42.26						
					15	0.69	40.93						
					16								
					17								
					18								
					19								
					20								
					21								
					22	0.85	36.21						
					23	0.80	38.24						
					24	0.81	37.12						
					25	0.77	38.83						
					26	0.76	37.17						
					27	0.73	37.19						
					28	0.77	36.20						
					29	0.73	36.37						
					30	0.68	42.28						

								Faecal samples				Temp	
Flock	Date	Co.	Farm	House	Age	Mean	Kurtosis	day21	day28	day35	Result	tmax	tmin
55	20.6.1	1	1	1	1			Neg	Pos	Pos	POS	21.4	11.3
					2								
					3								
					4								
					5								
					6								
					7								
					8	0.71	28.53						
					9	0.73	29.11						
					10	0.75	30.27						
					11	0.76	30.66						
					12	0.77	31.22						
					13	0.77	30.75						
					14	0.77	30.49						
					15	0.78	31.11						
					16	0.77	30.03						
					17	0.75	31.77						
					18	0.75	30.84						
					19	0.73	32.40						
					20	0.69	32.50						
					21	0.70	33.07						
					22								

					23								
					24								
					25								
					26								
					27								
					28								
					29								
					30								

								Faecal samples				Temp	
Flock	Date	Co.	Farm	House	Age	Mean	Kurtosis	day21	day28	day35	Result	tmax	tmin
57	20.6.1	1	1	3	1			Neg	Pos	Pos	POS	21.4	11.3
					2								
					3								
					4								
					5								
					6								
					7								
					8	0.72	21.59						
					9	0.71	22.25						
					10	0.69	24.30						
					11	0.69	24.05						
					12	0.67	24.47						
					13	0.67	25.41						
					14	0.67	25.77						
					15	0.68	25.94						
					16	0.66	26.61						
					17								
					18								
					19								
					20								
					21								
					22								
					23								
					24								
					25								
					26								
					27								
					28								
					29								
					30								

								Faecal samples				Temp	
Flock	Date	Co.	Farm	House	Age	Mean	Kurtosis	day21	day28	day35	Result	tmax	tmin
58	6.8.14	1	1	1	1			Neg	Pos	Pos	POS	19.8	11.3
					2	0.76	15.51						

					3	0.74	17.21						
					4	0.73	19.62						
					5	0.71	21.62						
					6	0.68	27.05						
					7	0.70	27.33						
					8	0.70	27.71						
					9	0.72	28.04						
					10	0.72	28.79						
					11	0.78	30.41						
					12	0.80	30.33						
					13	0.81	31.35						
					14	0.82	32.76						
					15	0.83	33.51						
					16	0.82	33.39						
					17	0.83	33.82						
					18	0.82	31.55						
					19	0.83	36.49						
					20	0.80	35.28						
					21	0.78	34.12						
					22	0.83	35.25						
					23	0.77	34.70						
					24	0.77	35.91						
					25	0.82	36.09						
					26	0.78	35.52						
					27	0.74	35.00						
					28	0.75	34.99						
					29								
					30								

								Faecal samples				Temp	
Flock	Date	Co.	Farm	House	Age	Mean	Kurtosis	day21	day28	day35	Result	tmax	tmin
61	5.8.14	1	1	4	1			Neg	Neg	Pos	POS	19.8	11.3
					2								
					3								
					4	0.62	21.17						
					5	0.65	21.82						
					6	0.63	26.00						
					7	0.62	27.25						
					8	0.64	27.76						
					9	0.66	27.09						
					10	0.65	27.61						
					11	0.65	28.45						
					12	0.69	24.42						
					13	0.70	29.38						
					14	0.66	30.38						
					15	0.64	29.96						

					16	0.64	31.43						
					17	0.65	30.34						
					18	0.63	32.11						
					19	0.65	32.59						
					20	0.65	32.48						
					21	0.65	32.94						
					22	0.66	33.60						
					23	0.63	34.53						
					24	0.62	33.84						
					25	0.67	34.90						
					26	0.72	36.25						
					27	0.68	34.72						
					28	0.70	33.08						
					29								
					30								

								Faecal samples					
Flock	Date	Co.	Farm	House	Age	Mean	Kurtosis	day21	day28	day35	Result	Temp	
62	5.8.14	1	1	5	1			Neg	Pos	Pos	POS	tmax	tmin
					2	0.70	11.48					19.8	11.3
					3	0.69	14.68						
					4	0.68	16.92						
					5	0.66	20.49						
					6	0.61	25.57						
					7	0.62	28.65						
					8	0.66	29.67						
					9	0.72	30.13						
					10	0.76	30.78						
					11	0.78	30.60						
					12								
					13								
					14								
					15								
					16								
					17								
					18								
					19								
					20								
					21								
					22								
					23								
					24								
					25								
					26								
					27								
					28								

					29								
					30								

								Faecal samples					
Flock	Date	Co.	Farm	House	Age	Mean	Kurtosis	day21	day28	day35	Result	Temp	
63	5.8.14	1	1	6	1			Neg	Pos	Pos	POS	tmax	tmin
					2							19.8	11.3
					3	0.83	16.71						
					4	0.79	19.26						
					5	0.77	22.94						
					6	0.74	25.80						
					7	0.73	28.53						
					8	0.76	28.75						
					9	0.79	29.13						
					10	0.80	28.77						
					11	0.81	29.61						
					12	0.81	30.61						
					13	0.81	30.72						
					14	0.82	30.77						
					15	0.83	31.64						
					16	0.87	32.74						
					17	0.87	32.68						
					18	0.82	32.64						
					19	0.82	31.95						
					20	0.81	31.64						
					21	0.83	32.37						
					22	0.79	32.65						
					23	0.75	32.10						
					24	0.75	30.43						
					25	0.72	31.51						
					26	0.75	31.79						
					27	0.78	32.99						
					28	0.81	32.66						
					29								
					30								

								Faecal samples					
Flock	Date	Co.	Farm	House	Age	Mean	Kurtosis	day21	day28	day35	Result	Temp	
64	23.9.1	1	1	1	1			Neg	Neg	Neg	NEG	tmax	tmin
					2	0.68	7.42					20.8	10.8
					3	0.66	8.21						
					4	0.66	10.93						
					5	0.64	12.02						
					6	0.63	16.59						
					7	0.62	19.65						
					8	0.62	22.48						

					9	0.63	24.35						
					10	0.64	26.82						
					11	0.64	25.48						
					12	0.64	27.38						
					13	0.65	27.30						
					14	0.66	29.51						
					15	0.67	30.38						
					16	0.67	28.44						
					17								
					18								
					19								
					20								
					21								
					22	0.65	30.27						
					23	0.68	28.54						
					24	0.66	30.69						
					25	0.68	28.58						
					26	0.69	29.97						
					27	0.70	29.06						
					28	0.70	27.77						
					29	0.68	28.41						
					30	0.69	27.26						

								Faecal samples					
Flock	Date	Co.	Farm	House	Age	Mean	Kurtosis	day21	day28	day35	Result	Temp	
65	23.9.1	2	1	2	1			Neg	Neg	Neg	NEG	tmax	tmin
					2	0.79	12.81					20.8	10.8
					3	0.75	16.38						
					4	0.74	16.20						
					5	0.73	19.26						
					6	0.67	23.17						
					7	0.64	23.72						
					8	0.69	25.96						
					9	0.71	27.16						
					10	0.72	27.80						
					11	0.74	26.51						
					12	0.69	28.86						
					13	0.68	28.41						
					14	0.68	28.20						
					15	0.70	28.24						
					16	0.68	28.41						
					17								
					18								
					19	0.66	31.35						
					20	0.65	30.48						
					21	0.64	30.67						

					22	0.66	29.29						
					23	0.64	30.32						
					24	0.67	29.69						
					25	0.64	29.80						
					26	0.65	30.60						
					27	0.69	31.34						
					28	0.71	29.20						
					29	0.72	28.88						
					30	0.70	28.92						

								Faecal samples					
Flock	Date	Co.	Farm	House	Age	Mean	Kurtosis	day21	day28	day35	Result	Temp	
66	23.9.1	1	1	3	1			Neg	Neg	Neg	NEG	tmax	tmin
					2	0.77	12.23					20.8	10.8
					3	0.75	11.49						
					4	0.72	13.76						
					5	0.69	15.57						
					6	0.64	20.96						
					7	0.63	22.53						
					8	0.65	24.15						
					9	0.69	26.96						
					10	0.74	27.00						
					11	0.77	26.07						
					12	0.77	27.44						
					13	0.79	28.07						
					14	0.76	27.40						
					15	0.75	29.78						
					16	0.76	28.99						
					17	0.72	32.25						
					18	0.70	30.01						
					19	0.69	31.51						
					20	0.66	32.16						
					21	0.68	31.29						
					22	0.68	31.52						
					23	0.69	31.37						
					24	0.65	31.30						
					25	0.64	31.56						
					26	0.61	31.76						
					27	0.65	31.75						
					28	0.68	31.49						
					29	0.72	28.22						
					30								

								Faecal samples					
Flock	Date	Co.	Farm	House	Age	Mean	Kurtosis	day21	day28	day35	Result	Temp	
67	23.9.1	1	1	4	1			Neg	Neg	Neg	NEG	tmax	tmin

					2	0.73	10.70					20.8	10.8
					3	0.72	11.17						
					4	0.71	13.17						
					5	0.70	15.73						
					6	0.66	22.69						
					7	0.67	23.87						
					8	0.68	24.17						
					9	0.71	27.60						
					10	0.74	29.31						
					11	0.78	28.79						
					12	0.80	30.20						
					13	0.80	31.56						
					14	0.79	31.80						
					15	0.80	31.59						
					16	0.78	33.61						
					17	0.71	31.68						
					18								
					19	1.13	12.05						
					20	0.66	31.84						
					21	0.65	30.30						
					22	0.64	31.68						
					23	0.67	29.58						
					24	0.64	33.23						
					25	0.65	32.05						
					26	0.63	32.76						
					27	0.66	33.67						
					28	0.67	32.91						
					29	0.68	32.64						
					30	0.69	31.91						

								Faecal samples					
Flock	Date	Co.	Farm	House	Age	Mean	Kurtosis	day21	day28	day35	Result	Temp	
69	23.9.1	1	1	6	1			Neg	Neg	Neg	NEG	tmax	tmin
					2	0.71	9.78					20.8	10.8
					3	0.70	9.03						
					4	0.67	10.26						
					5	0.66	12.04						
					6	0.63	15.18						
					7	0.62	18.11						
					8	0.62	18.73						
					9	0.64	25.04						
					10	0.66	25.57						
					11	0.68	26.55						
					12	0.67	27.38						
					13	0.68	26.86						
					14	0.66	27.50						

					15	0.70	30.65						
					16	0.70	32.10						
					17								
					18								
					19								
					20								
					21								
					22								
					23								
					24								
					25								
					26								
					27								
					28								
					29								
					30								
